# US Public Attitudes Toward COVID-19 Vaccine Mandates

**DOI:** 10.1001/jamanetworkopen.2020.33324

**Published:** 2020-12-18

**Authors:** Emily A. Largent, Govind Persad, Samantha Sangenito, Aaron Glickman, Connor Boyle, Ezekiel J. Emanuel

**Affiliations:** 1 Department of Medical Ethics and Health Policy, University of Pennsylvania Perelman School of Medicine, Philadelphia; 2 Leonard Davis Institute of Health Economics, Philadelphia, Pennsylvania; 3 University of Denver Sturm College of Law, Denver, Colorado; 4 Fels Institute of Government, University of Pennsylvania, Philadelphia; 5 Penn Program on Opinion Research and Election Studies, University of Pennsylvania, Philadelphia

## Abstract

This survey study assesses the acceptability of coronavirus disease 2019 (COVID-19) vaccine mandates among members of the US public.

## Introduction

Ending the coronavirus disease 2019 (COVID-19) pandemic through vaccination will require sufficient uptake, possibly through mandatory vaccination. At present, certain vaccines are required for children to attend school.^1^ Although vaccine mandates for adults are legal, they have generally been applied narrowly to select groups, such as health care workers, rather than broadly enforced.^2^ We surveyed the US public to assess acceptability of COVID-19 vaccine mandates.

## Methods

The University of Pennsylvania institutional review board exempted this survey study because the survey was anonymous and the information was recorded in such a way that the identity of the respondents cannot be ascertained. The survey followed proprietary Gallup guidelines.

Results are based on a Gallup Panel web study completed between September 14 and 27, 2020, by 2730 consenting US adults, aged 18 years and older. Participants consented through the survey website. The survey response rate was 39% (American Association for Public Opinion Research RR1). The Gallup Panel is a well-established, probability-based panel. Respondents were asked about the acceptability of states requiring adults and children and employers requiring employees to “get the COVID-19 vaccine (unless they have a medical reason not to be vaccinated).”

Descriptive statistics were calculated using Gallup-provided survey weights to generate nationally representative estimates. Respondents’ answers were compared using χ^2^ tests accounting for survey weights. Statistical significance was set at α = .05 for 2-tailed tests. Analyses were conducted using R statistical software version 4.0.2 (R Project for Statistical Computing).

## Results

The sample was weighted to be demographically representative of the US population (Table). Overall, 61.4% (95% CI, 60.0%-63.0%) of respondents indicated they would likely get a COVID-19 vaccine. Republicans and Independents were, however, significantly less likely to get vaccinated than Democrats (Republicans, 44.3% [95% CI, 41.7%-46.8%]; Independents, 58.4% [95% CI, 55.5%-61.1%]; Democrats, 76.6% [95% CI, 74.7%-78.5%]), and Black respondents were significantly less likely than non-Black respondents to get vaccinated (43.6% [95% CI, 39.2%-48.2%] vs 63.7% [95% CI, 62.3%-65.2%]).

**Table.** Respondent Demographic Characteristics

Characteristic	Respondents	
	Unweighted No. (%)	Weighted No. (%)
Gender		
Female	1250 (45.9)	1396 (51.2)
Male	1474 (54.1)	1328 (48.8)
Race/ethnicity		
Asian	47 (1.7)	19 (0.6)
Black	118 (4.3)	307 (11.3)
Hispanic	148 (5.4)	405 (14.9)
White	2385 (87.7)	1984 (72.9)
Other	22 (0.8)	5 (0.8)
Political affiliation		
Democrat	1121 (41.4)	1099 (40.5)
Independent	678 (25.0)	675 (24.9)
Republican	829 (30.6)	825 (30.4)
Other	80 (3.0)	114 (4.2)
Education		
Less than a bachelor’s degree	1155 (42.4)	1795 (65.9)
Bachelor’s degree or higher	1569 (57.6)	929 (34.1)

Nearly one-half (48.6%; 95% CI, 44.8%-53.0%) of respondents regarded requiring COVID-19 vaccination for children attending school as acceptable or very acceptable (hereafter, *acceptable*), and 38.4% (95% CI, 34.6%-42.0%) regarded it as unacceptable or very unacceptable (hereafter, *unacceptable*) (Figure). Although 40.9% (95% CI, 37.2%-45.0%) of respondents found state mandates for adults acceptable, 44.9% (95% CI, 41.0%-49.0%) found them unacceptable. Compared with state mandates for adults, slightly more respondents (47.7%; 95% CI, 43.8%-52.0%) found employer-enforced employee mandates acceptable, whereas 38.1% (95% CI, 34.4%-42.0%) found them unacceptable.

**Figure.**
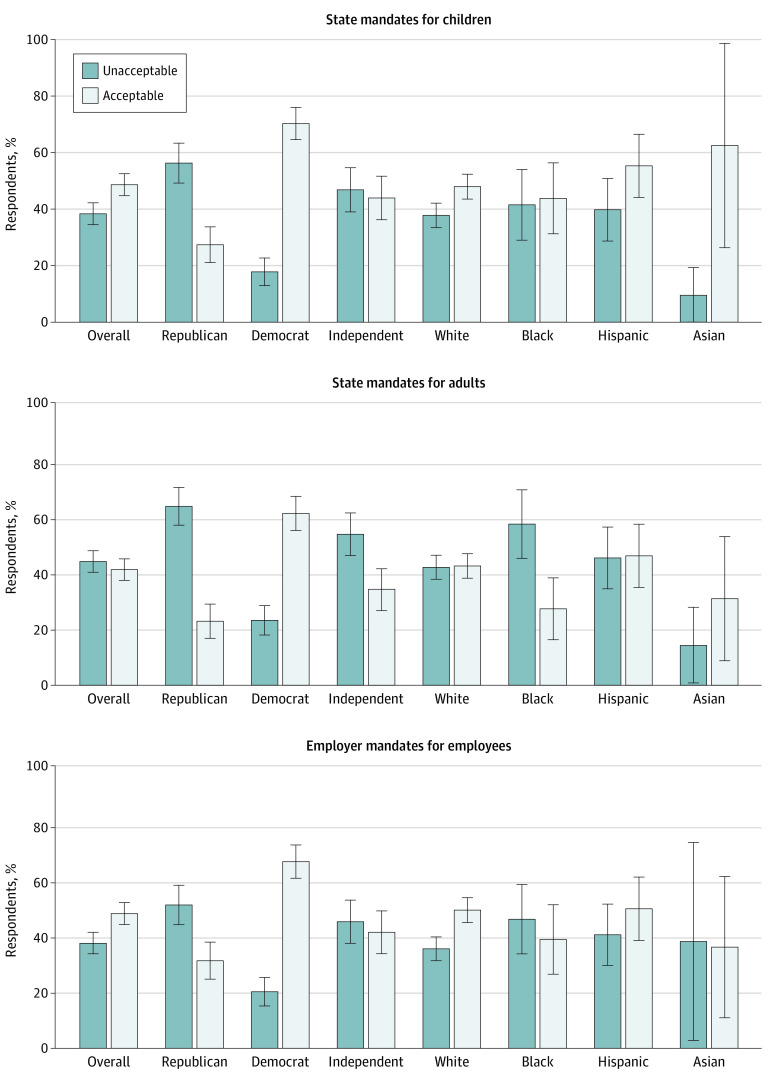
Acceptability of Mandates Error bars indicate 95% CIs.

Individuals likely to get a COVID-19 vaccine accepted mandates at higher rates than those unlikely to do so (mandates for children, 73.6% [95% CI, 68.5%-78.1%] vs 23.7% [95% CI, 19.4%-28.7%]; mandates for adults, 65.0% [95% CI, 59.7%-69.9%] vs 17.3% [95% CI, 13.6%-21.7%]; mandates for employees, 72.5% [95% CI, 67.3%-77.1%] vs 22.9% [95% CI, 18.6%-27.8%]). Democrats were likelier than Republicans and Independents to accept state mandates for children (Republicans, 27.4% [95% CI, 21.5%-34.2%]; Independents, 44.0% (95% CI, 36.5%-51.7%); Democrats, 70.2% [95% CI, 64.3%-75.7%]) and adults (Republicans, 22.6% [95% CI, 17.1%-29.3%]; Independents, 34.0% [95% CI, 27.1%-41.5%]; Democrats, 60.8% [95% CI, 54.6%-66.6%]) and employer-enforced employee mandates (Republicans, 31.0% [95% CI, 24.8%-37.9%]; Independents, 41.0% [95% CI, 33.7%-48.8%]; Democrats, 66.0% [95% CI, 59.9%-71.7%]). Compared with non-Black respondents, fewer Black respondents accepted state mandates for adults (42.7% [95% CI, 38.7%-46.8%] vs 27.0% [95% CI, 17.5%-39.2%]), and more found them unacceptable (43.1% [95% CI, 39.1%-47.2%] vs 58.4% [95% CI, 45.7%-70.1%]). Respondents with a bachelor’s degree or higher were likelier to find mandates acceptable than those without (mandates for children, 66.0% [95% CI, 60.1%-71.4%] vs 39.7% [95% CI, 34.9%-44.6%]; mandates for adults, 56.4% [95% CI, 50.4%-62.2%] vs 32.9% [95% CI, 28.3%-37.7%]; mandates for employees, 62.4% [95% CI, 56.5%-68.0%] vs 39.9% [95% CI, 35.2%-44.9%]). No gender differences were observed.

## Discussion

Vaccine mandates have drawn attention because of growing concerns that voluntary COVID-19 vaccination rates will be insufficient to stem transmission.^3,4^ Consistent with prior research,^5^ we found that demographic characteristics and partisanship were associated with self-reported likelihood of COVID-19 vaccination. Demographic characteristics and partisanship were also associated with acceptance of COVID-19 vaccine mandates. This suggests that in some states or localities, COVID-19 vaccine mandates—particularly for adults—may be ineffective or, worse, prompt backlash.^6^ Employer-enforced employee mandates did not garner majority acceptance; however, acceptability exceeded unacceptability, suggesting a potential role for employers to increase COVID-19 vaccine uptake, particularly among key groups such as frontline workers.

A limitation of this study is that respondents described the acceptability of hypothetical COVID-19 vaccine mandates. Responses may differ as efficacy and safety evidence for actual COVID-19 vaccines develop and if perceptions of pandemic politicization change.

Public health efforts aimed at making COVID-19 vaccines accessible and improving uptake should continue before considering mandates. Mandates should be used only if COVID-19 continues to be inadequately contained and voluntary vaccine uptake is insufficient.
